# The pearls and pitfalls of setting high-quality multiple choice questions for clinical medicine

**DOI:** 10.4102/safp.v65i1.5726

**Published:** 2023-05-29

**Authors:** Mergan Naidoo

**Affiliations:** 1 College of Family Physicians of South Africa, Discipline of Family Medicine, School of Nursing and Public Health, University of KwaZulu-Natal, Durban, South Africa

**Keywords:** multiple choice questions, MCQs, single best answer questions, blueprinting, post hoc analysis

## Abstract

Multiple choice question (MCQ) examinations have become extremely popular for testing applied knowledge in the basic and clinical sciences. When setting MCQ examinations, assessors need to understand the measures that improve validity and reliability so that the examination accurately reflects the candidate’s ability. This continuing medical education unit will cover the essentials of blueprinting an exam, constructing high-quality MCQs and post hoc vetting of the exam. It is hoped that academics involved in assessments use the content provided to improve their skills in setting high-quality MCQs.

## Introduction

Multiple choice examinations have become extremely popular for testing applied knowledge in the basic and clinical sciences. When setting any examination, it is essential to establish important issues that underpin the test. Areas to consider include whether the assessment is formative or summative and whether a suitable blueprint exists. The examiner must also consider issues of validity and reliability. Validity speaks to whether the test measures what it is intended for and is aligned with the purpose of the test.^1^ Although many forms of validity exist, important ones to consider are the content, construct and criterion-related validity. Reliability speaks to consistency and is often measured using various psychometric parameters often evaluated in the post hoc analysis.^2^ The number of items and the test duration also contribute to the validity and reliability.^3^ Generally, 3 h to 4 h of testing and having 100 or more test items are associated with good reliability.^3^ This continuing medical education unit will cover essential elements of setting multiple choice questions (MCQs), blueprinting, constructing MCQs, and briefly delve into the use of psychometrics in the post hoc analysis and standard setting.

## Blueprinting

Blueprinting is the process that ensures a match between the curriculum and the assessment system. It answers what you need to measure, how you plan to assess the learning and the importance of each area to be tested.^4^ Ideally, the entire module, course or programme’s learning outcomes should be blueprinted against the assessment method. The advantage of MCQs is that it allows for testing a broader curriculum selection. However, the examining team needs to define the learning areas and content to be tested in this paper. Also, it is crucial to establish at what level Bloom’s (or an alternative) taxonomy is acceptable. The team may find it acceptable to have 10% of the paper set at an understanding level, 80% at an application level and 10% at an analysis level. After you decide what you are testing, you need to decide on the relative importance of the content to be tested. The test blueprint will include the knowledge, skills, and attitudes to be tested, specific topics to be covered and the number of questions per topic.^4^ A useful method of working out the number of questions in clinical medicine is to calculate the ‘impact factor’ (IF) by multiplying the frequency of occurrence of a condition and the implication to health. The total IF is then calculated for the entire exam, and the topic IF is divided by the total IF and multiplied by the total questions for a paper. An example is illustrated in Table 1. We need to set 20 questions for the fellowship exam in family medicine. The section blueprint is valuable for the examiner constructing the question from this section. The frequency of occurrence used in the table is divided into common occurrences (daily to weekly) and given a score of three, frequent occurrences (every few weeks up to 3 months) and given a score of two and rare occurrences (more than 3 months) and given a score of one. The implication is rated as essential (immediately life-threatening) and given a score of four, important (life-threatening but delayed) and given a score of three, additional (organ or limb-threatening) and given a score of two and nice to know (trivial) and given a score of one.

**TABLE 1:** Allocating the number of questions for a topic within a section.

Surgery content	Frequency of occurrence	Implication	IF	Number of MCQs
Abscess	3	2	6	6/163*20 = 1
Ingrowing toenails	2	1	2	0.2
Peritonsillar abscess	1	4	4	0.4
Varicose veins	3	1	3	0.4
Peripheral vascular disease	3	2	6	**1.0**
Aneurysms	1	4	4	0.4
Acute abdomen	2	4	8	**1.0**
Appendicitis	2	3	6	**1.0**
Gall stones	2	3	6	**1.0**
Pancreatitis	3	3	9	**2.0**
Dysphagia	2	2	4	0.2
GI bleeding	3	4	12	**2.0**
GI cancer	2	3	6	0.4
Bowel obstruction	1	3	3	0.2
Hernias	3	2	6	**1.0**
Inflammatory bowel disease	1	3	3	0.4
Diverticular disease	2	2	4	0.4
Haemorrhoids	3	1	3	0.4
Perianal haematoma	2	1	2	0.2
Paraphimosis	2	2	4	0.4
Hydrocoele	2	1	2	0.2
Calculi	3	2	6	**1.0**
Prostatic disease	3	2	6	**1.0**
Testicular disease	1	2	2	0.2
Haematuria	3	2	6	**1.0**
Urinary tract cancers	1	3	3	0.4
Incontinence	3	2	6	**1.0**
Lymphadenopathy	3	2	6	**1.0**
Neck lumps	2	2	4	0.4
Thyroid masses	3	3	9	**2.0**
Plastic surgery	3	1	3	0.4
Male medical circumcision	3	3	9	**2.0**

**Total impact factor score**	**-**	**-**	**163**	-

Note: The data in bold are where you need to choose your questions.

MCQs, multiple choice questions; IF, impact factor.

## Constructing multiple choice questions

The construction of MCQs is based on the guidelines from the National Board of Clinical Examiners (NBME).^5^ It is our practice to use single best answer (SBA) and extended matching questions (EMQs) in our tests. The question must focus on common and essential conditions and assess Bloom’s taxonomy’s higher levels. The MCQs must be checked for alignment with learning outcomes and curriculum content. Pre-test vetting by an assessment committee is especially important for high-stakes examinations. The scenario must always have a good story (vignette), including age, gender and setting, as context is important. Do not provide the diagnosis and avoid cues that may alert test-savvy candidates. The vignette should have specific information that points to the most correct option being selected. The lead-in should be focused, closed, and clear and asked as a question. The candidates must not select the correct response based on the lead-in alone. All the options must be homogenous, that is, all investigations or all management options, and must be plausible. A review of the MCQ is critical to identify and remove technical flaws that add irrelevant difficulty. The following steps are suggested for setting an SBA MCQ:

- Decide on a common and important topic that you would like to test.
- Write down the correct option.
- Add all possible plausible distractors, even if they are more than those required.
- Write the lead-in as a question.
- Add the vignette containing all the essential information.
- Include the specific information that will allow the candidate to select the correct option.
- Check if all distractors are still plausible and select the correct number of distractors.
- Correct grammatical and technical flaws.
- Cover the options and see if you can answer the question.

**FIGURE 1:**
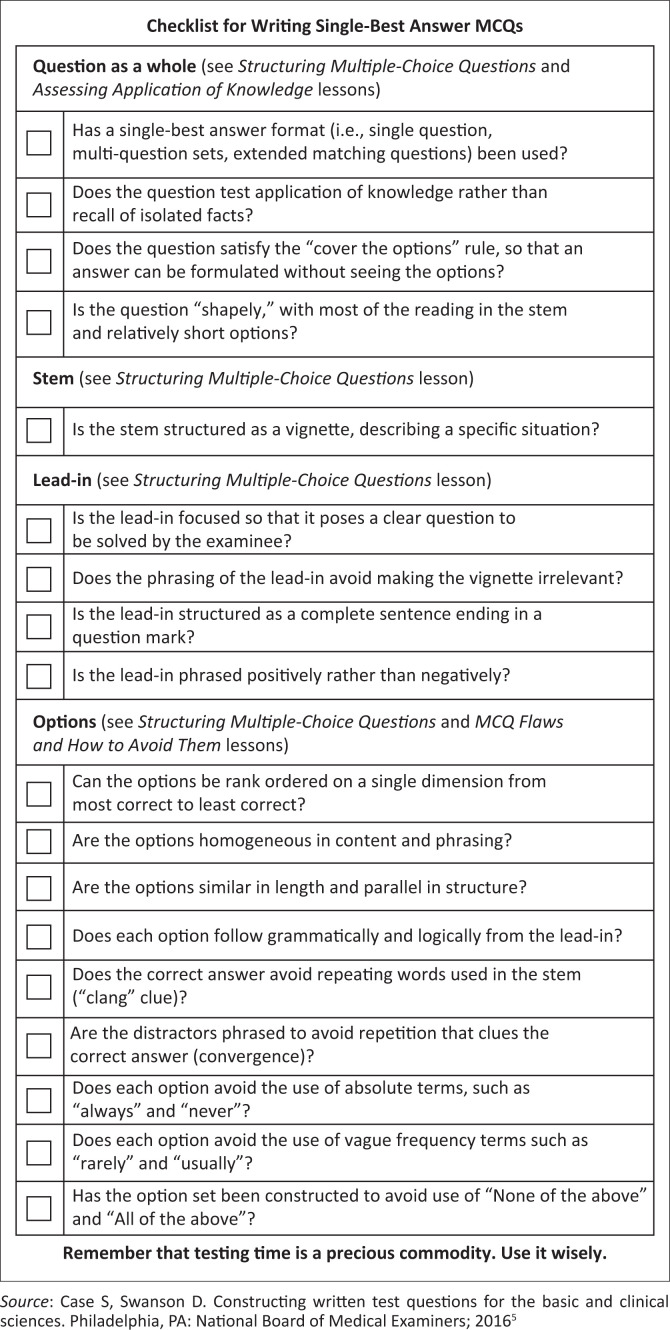
Checklist for writing single best answer questions.

The NBME has provided a helpful checklist for assessors, which is shown in Figure 1. Examiners must use these if they are not familiar with the process.

**FIGURE 2:**
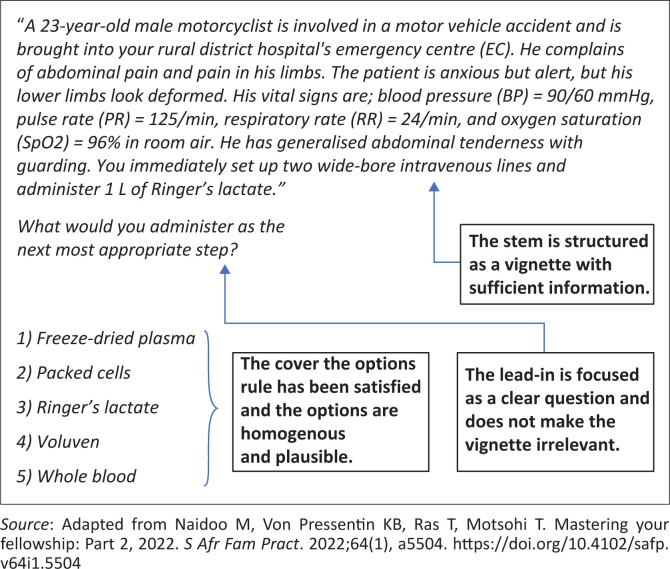
An example of a single best answer multiple choice question.

Figure 2 reflects an example of an SBA published in the Mastering Your Fellowship^6^ series in 2022.

Extended matching questions benefit primary care assessments, especially for undifferentiated conditions. The recommended steps are as follows:^7^

- Decide on a theme, for example, diagnosis, investigations, management based on a test blueprint, that is, abdominal pain.
- Develop the options list aiming for at least three plausible options per stem.
- Extended matching questions are best constructed by a team of assessors who can be given the option of writing a set of vignettes (stems) after the options have been agreed on.
- Now write the stem excluding the lead-in, as listed in the given SBA construction.
- Put the options and stems together and include an appropriate lead-in.
- Peer review as for the given SBAs (Steps 7–9)

The use of images such as X-rays, electrocardiograms (ECGs) and pictures of clinical presentations is also helpful in augmenting the MCQ. Testing time needs to be decided on by the assessment committee, but generally, between 90 s and 120 s are recommended per item. After the questions have been finalised, a system of coding the questions should be implemented, and the questions should be banked for easy retrieval. Multiple-choice questions must be reviewed every 2 years. The performance statistics of questions are reviewed as part of the post hoc analysis.^5,7^

True and false formats are not recommended for assessments in clinical medicine. Some of the pitfalls involved in writing MCQs are the following:

- Flaws related to test-savvy candidates include grammatical clues, logical cues, absolute terms, long options, clang options and convergence strategies.
- Flaws related to irrelevant difficulties include ordering information disjointly and not stating numerical data consistently.
- Using vague terms and options, non-parallel language options and using none of the above or all of the above options poses problems with question design.^7^

## Post hoc analysis

The role of the assessment team is not complete until they have assessed the post-test psychometrics. Various metrics exist, often measured by software programmes to aid the calculation of psychometrics. The coefficient alpha and Kuder-Richardson formulas help measure reliability of the examination. These intraclass correlations (generalisability coefficients) provide information on the expected strength of the relationship between the scores observed on this test and the scores observed on tests covering similar but not identical content.^7^ Values approximating one indicate that retesting is likely to yield similar scores. Most centres aim for scores over 0.8 in high-stakes examinations. Another useful reliability measure is the standard error of measurement (SEM). The SEM is the difference between a candidate’s ability and test score. Suppose the same test was taken repeatedly, with no change in the level of knowledge and preparation. In that case, some results may be higher or lower than the score that accurately reflects their ability.^7^ The ‘true’ score resides between the candidate’s score and an SEM. The smaller the SEM (ideally less than 4%), the greater the degree of precision that the test accurately reflects the actual ability of the candidates.

The next step in the post hoc analysis is item analysis. Ideally, these would be useful when 25 or more candidates take the test. This analysis should include which items do not meet the minimum acceptable criteria. The reasons for poor psychometrics include content not being taught or understood, ambiguous question wording, or the wrong answer being keyed in. A panel of experts then reviews such questions. These questions may be removed, or the correct answer may be keyed in, and the question remarked. The item analysis should include the number of candidates who took the test, the number who answered the item and which options were selected. The report should also have the item difficulty (number of candidates who got the question correct) and the item discrimination. The discriminatory index (DI) is the degree to which questions discriminate between participants who know the material well and those who do not. The DI is measured using one of the following measures:

- High-low discrimination: Subtract the percentage of low-scoring participants who got the item correct from the percentage of high-scoring participants who got the item right.
- Item-total correlation coefficient: A point-biserial correlation between candidate test scores and items scores.

The preferred range for the easiness index (also called the facility index) and the DI is 0.3–0.8. Negative DIs are always problematic and need discussion among expert reviewers. Questions scoring outside this range need to be reviewed by the expert panel and decisions should be made on what action should be taken.^7,8^ The flaws of questions with low easiness indices include obscure content or content not taught, poorly worded or confusing items, items delivered at the end of a timed test, questions wrongly scored and two choices that are both correct. Reasons for questions that are too easy include well-known content, an item that has been exposed and shared, clues in the item as to what the correct answer is and poor distractors. Low DIs may be because of the item being too easy or too hard, the correct answer is awry, there is more than one correct answer, the question is ambiguous or poorly written, the high-performing candidates are overthinking it, the question is measuring a different construct compared with other items, and there is a small sample size.

## Standard setting

In high-stakes postgraduate medicine examinations in South Africa, standard setting has become routine practice. Standard-setting is used to determine what examination score best represents a satisfactory level of competency in the discipline.^9,10^ Various standard-setting methods exist. The most popular method used in South Africa for the MCQ examination is the Angoff or modified Angoff^11^ method of standard setting used for tests with 50 or fewer candidates. The Cohen or modified Cohen^12^ method of standard setting is more popular for larger examinations of more than 50 candidates. The standard setting methods will not be described in this manuscript, but I suggest to read this manuscript’s references for those interested.

## Conclusion

Writing MCQs is a skill, and like all skills, it requires practice and mentorship. The MCQs have become extremely popular for knowledge-based exams, especially during the coronavirus disease 2019 (COVID-19) pandemic. Understanding the blueprint, the question construction, and the use of the post hoc analysis will help deliver valid and reliable examinations.
